# EPLIN, a Putative Tumour Suppressor in Colorectal Cancer, Implications in Drug Resistance

**DOI:** 10.3390/ijms232315232

**Published:** 2022-12-03

**Authors:** Jianyuan Zeng, Andrew J. Sanders, Lin Ye, Rachel Hargest, Fiona Ruge, Wen G. Jiang

**Affiliations:** 1 School of Medicine, Cardiff University, Henry Wellcome Building, Cardiff CF14 4XN, UK; 2 School of Natural and Social Science, University of Gloucestershire, Francis Close Hall, Swindon Road, Cheltenham GL50 4AZ, UK

**Keywords:** EPLIN, colorectal cancer, HSP60, Her2, drug resistance

## Abstract

Colorectal cancer is a serious threat to human health. Poor prognosis and frequently reported drug resistance urges research into novel biomarkers and mechanisms to aid in the understanding of the development and progression of colorectal cancer and to optimise therapeutic strategies. In the current study, we investigated the roles of a putative tumour suppressor, EPLIN, in colorectal cancer. Our clinical colorectal cancer cohort and online databases revealed a downregulation of EPLIN in colorectal cancer tissues compared with normal tissues. The reduced expression of EPLIN was associated with poor clinical outcomes of patients. In vitro cellular function assays showed that EPLIN elicited an inhibitory effect on cellular growth, adhesion, migration and invasion. Utilising a protein microarray on protein samples from normal and tumour patient tissues suggested HSP60, Her2 and other signalling events were novel potential interacting partners of EPLIN. It was further revealed that EPLIN and HSP60 were negative regulators of Her2 in colorectal cancer cells. The clinical cohort also demonstrated that expression of HSP60 and Her2 affected clinical outcomes, but most interestingly the combination of EPLIN, HSP60 and Her2 was able to identify patients with the most unfavourable clinical outcome by independently predicting patient overall survival and disease free survival. Furthermore, EPLIN and HSP60 exhibited potential to regulate cellular response to chemotherapeutic and EGFR/Her2 targeted therapeutic agents. In conclusion, EPLIN is an important prognostic factor for patients with colon cancer and reduced EPLIN in CRC contributes to aggressive traits of CRC cells and their responses to chemotherapeutic drugs. Collectively, EPLIN is a pivotal factor for the development and progression of colorectal cancer and has important clinical and therapeutic values in this cancer type.

## 1. Introduction

Colorectal cancer is one of the leading cancers globally and is particularly challenging in Western countries. In high/very high human development index (HDI) countries, the age-standardised incidence of colorectal cancer came second at 20 per 100,000 females population after breast cancer in females and third at 29.0 per 100,000 male population after prostate and lung cancers [1]. The death rate of colorectal cancer in HDI countries ranked third in males and females alike [1]. Collectively, this represents 1.9 milion new colorectal cases and more than 930,000 death from colorectal cancer globally. In the UK where the present study is conducted, colorectal cancer ranked the 4th most common cancer type and 2nd most common cause of cancer death [2]. Thus, colorectal cancer (CRC) has become a heavy burden to public health. Despite a stable overall incidence rate and steadily improving survival rates in recent years, there is an increasing trend of early-onset CRC cases and challenging 5-year survival rates in more aggressive stages [2]. As one of the major treatments of CRC apart from surgery, chemotherapy remains important in treating CRC patients and has been delivered as single drug treatment such as 5-Fluorouracil (5-FU), Oxaliplatin, Docetaxel, Irinotecan, etc. and in combinational regimes such as OLFOX (5-FU, leucovorin (LV) and Oxaliplatin), Capeox (Capecitabine with Oxaliplatin), FOLFIRI (5-FU, LV with Irinotecan), CAPIRI (Capecitabine with Irinotecan), etc. [3]. However, therapeutic resistances to such agents are also frequently reported and are linked with worsened prognosis of the patients. Aberration in genes/translation factors and related signalling events may contribute to such resistances [4,5,6] as well as the development and progression of CRC [7,8]. Therefore, research focused on identifying novel markers to shed light on therapeutic strategies have become more and more urgent.

Encoded by the *LIMA1* gene, Epithelial Protein Lost In Neoplasm (EPLIN) was initially discovered to be downregulated in oral cancer [9,10]. EPLIN has been reported to be downregulated in multiple cancer types including breast cancer [11], prostate cancer [12,13,14], lung cancer [15] and ovarian cancer [16]. The downregulation of EPLIN links to poor prognosis of such cancer patients [11,13,15,16]. EPLIN has also been demonstrated to regulates cellular functions such as adhesion, growth, migration and invasion, essential to the aggressive phenotype of cancer cells [11,12,13,14,15,17]. To achieve its cellular impacts, EPLIN can be phosphorylated by extracellular signal-regulated kinase 1/2 (ERK1/2) upon activation of platelet derived growth factor (PDGF), subsequently leading to upregulation of β-catenin and Zinc Finger E-Box Binding Homeobox 1 (ZEB1) and promote the epithelial mesenchymal transition (EMT) process [12,18]. Meanwhile, EPLIN can be regulated by p53 family members to lessen the invasiveness of cancer cells [19,20]. Moreover, EPLIN is involved in a carcinogenetic progress namely apical elimination by interacting with plectin and paxillin [21]. 

Hence, EPLIN has been demonstrated to act as a potential novel tumour suppressor. In the present study, we explored the role of EPLIN in CRC in clinical cohorts and in vitro assays. Novel interacting partners of EPLIN were also investigated for their influence on clinical outcomes of CRC patients and impact on drug resistance to chemotherapeutic and targeted therapeutic agents. 

## 2. Results

### 2.1. Downregulation of EPLIN Links to Poor Prognosis in CRC Patients

**Figure 1:**
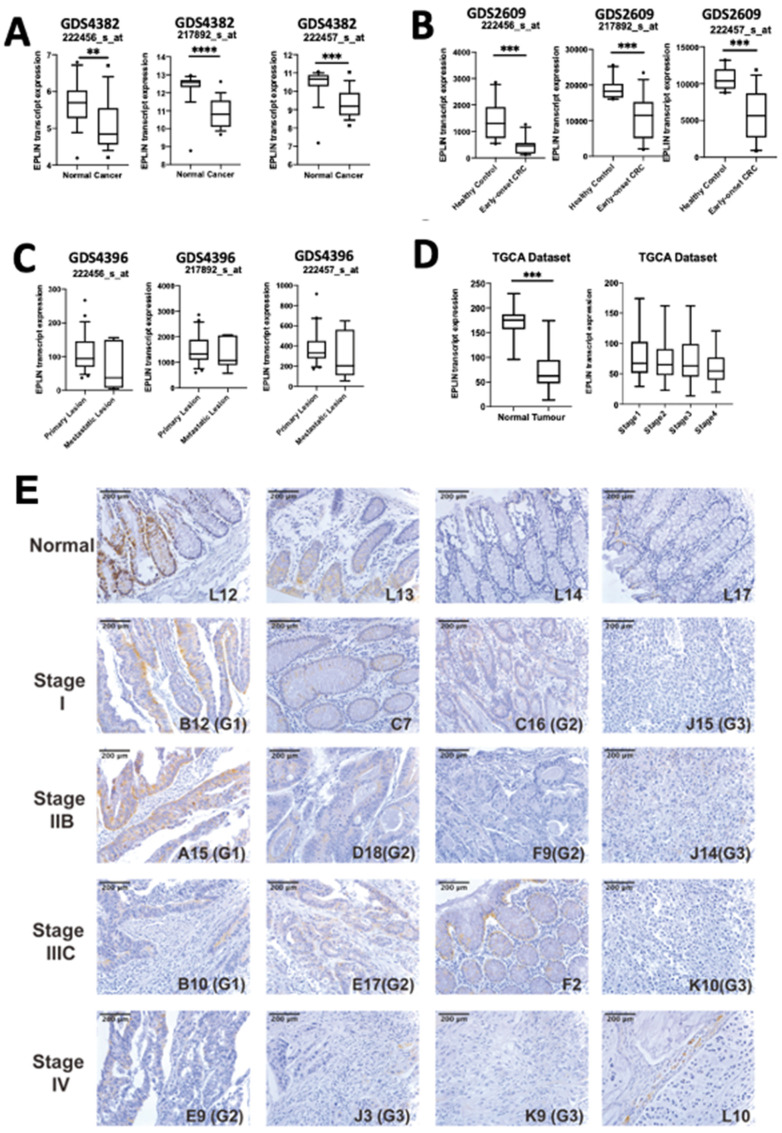

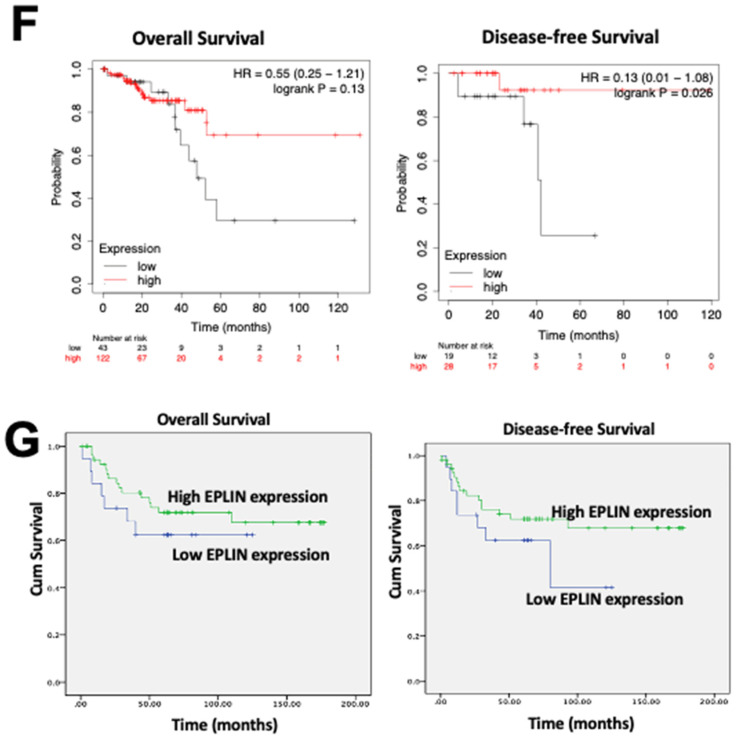
Expression of EPLIN in clinical CRC. (**A**) Expression of EPLIN in GEO GDS4382 dataset (normal: *n* = 17; cancer: *n* = 17). (**B**) transcript expression of EPLIN in GEO GDS2609 dataset (healthy control: *n* = 10; Early-onset CRC: *n* = 12). (**C**) Expression of EPLIN in GEO GDS4396 dataset (metastatic lesions: *n* = 6; primary lesions: *n* = 23). Box plot data shown as median expression, Q1 and Q3 value, whiskers represent 5th and 95th percentiles with outliers shown. EPLIN was detected by three different probes, 222456_s_at, 222457_s_at and 217892_s_at. (**D**) Analysis of EPLIN expression in colon adenocarcinoma in TCGA database. Box plot data shown as median expression, Q1 and Q3 value, whiskers represent 5th and 95th percentiles with outliers shown. (**E**) Analysis of EPLIN staining in the colorectal cancer TMA (CO2161a). (**F**) Survival curves of OS and DFS by probing EPLIN in the rectum adenocarcinoma on the KM plotter platform. (**G**). Implication of EPLIN on patient’s OS and DFS in the clinical cohort. * represents *p* < 0.05, ** represents *p* < 0.01, *** represents *p* < 0.001, **** represents *p* < 0.0001.

We first analysed the transcript expression of EPLIN in a local CRC clinical cohort (Table 1) and CRC public online datasets (Figure 1).

A CRC clinical cohort (*n* = 174) was employed in this study to explore the transcript expression profile of EPLIN, via qPCR, in comparison with patient’s clinical and pathological information (Table 1). The highlight significant finding of the cohort was that median transcript expression of EPLIN was diminished in tumour samples when compared to normal ones (*p* < 0.01). Besides, median transcript expression of EPLIN in invasive samples (median = 182, *n* = 26) was higher than it in non-invasive samples (median = 17.2, *n* = 50) (*p* = 0.0323). There were no significant differences in analysing other pathological information in the clinical cohort. 

Three CRC datasets from GEO database were employed, GDS4382 (*n* = 34; Figure 1A) [22], GDS2609 (*n* = 22; Figure 1B) [23] and GDS4396 (*n* = 29; Figure 1C) [24]. Three individual probes which target EPLIN (222456_s_at, 222457_s_at and 217892_s_at) were also applied to each dataset to assess its expression profile. In GDS4382, which contains normal and tumour samples, median transcript expression of EPLIN was significantly downregulated in cancer tissues compared with normal tissues (222456_s_at, *p* < 0.01; 222457_s_at and 217892_s_at, *p* < 0.001) (Figure 1A). Similarly, median transcript expression of EPLIN in early-onset CRC samples was also downregulated significantly when compared with healthy control (*p* < 0.001 for all probes) (Figure 1B). Although the trend of downregulation of EPLIN was present in metastatic lesions when compared with primary lesion, no statistical significance was noted (Figure 1C). Exploration of colon adenocarcinoma TCGA dataset via UALCAN platform [25] also revealed similar results (Figure 1D), in which median transcript expression of EPLIN was downregulated significantly in tumour samples when compared to normal ones (*p* < 0.001) while a non-significant downregulated trend could be observed in tumours of more aggressive stages.

In addition to assessing EPLIN’s transcript expression, we also explored the protein expression of EPLIN in CRC tissues by carrying out immunohistochemical staining on a CRC TMA (Table 2 and Figure 1E). The staining of EPLIN was generally stronger in normal tissues when compared to tumour tissues (Figure 1E). As shown in Table 2, more tumour tissues showed negative to weak EPLIN staining in adenocarcinoma (59.4%), mucinous adenocarcinoma (80%) and signet ring cell carcinoma tissues (2 of 3) than normal tissues (50%). Although chi-square did not return statistical significance (*p* > 0.05, all tumour tissues versus normal tissue group), the trend of weaker staining of EPLIN in tumour samples can been observed. Additionally, EPLIN intensity was found to decease in more aggressive stages and grades. Firstly, around 55.6% of tissues in Stage I displayed negative to weak staining of EPLIN, while such staining was 60.9% in Stage II, 68.6% in Stage III and 75% in Stage IV. Secondly, 51.5% of tissues in Grade-1 displayed negative to weak staining of EPLIN, while it was 66.3% in Grade-2 and 67.3% in Grade-3. Although overall chi-square test among groups did not result in significance, the trend of weaker staining of EPLIN in more aggressive tissues can be observed. 

Hence, we demonstrated that EPLIN was downregulated in CRC when compared with normal samples at both transcript and protein levels. Although limited evidences were shown that EPLIN expression was related to metastatic CRC, a larger cohort is required for further exploration.

### 2.2. Downregulation of EPLIN Links to Poor Prognosis in CRC Patients

As Figure 1G shows, patients with low levels of EPLIN had worse overall survival (OS) and disease-free survival (DFS) than those with high level of EPLIN (OS: high level: mean = 132.1 months; low level: mean = 84.7 months. DFS: high level: mean = 129.4 months; low level: mean = 74.2 months), though neither of these reached statistical significance (OS: *p* = 0.34; RFS: *p* = 0.21). 

Such relationship was also investigated using online datasets. By analysing available rectal adenocarcinoma datasets on Kaplan-Meier Plotter (www.kmplot.com, accessed on 1 October 2022), a similar result was revealed (Figure 1F). Patients with low level of EPLIN tended to have a worse OS compared to those with high level of EPLIN, although no statistical significance was noted. Interestingly, those with low level of EPLIN had a significant worse DFS than high level group (*p* = 0.023). Overall, downregulation of EPLIN indicates a worse clinical outcome in CRC patients. 

### 2.3. EPLIN Regulates Cellular Functions in CRC Cells

**Figure 2:**
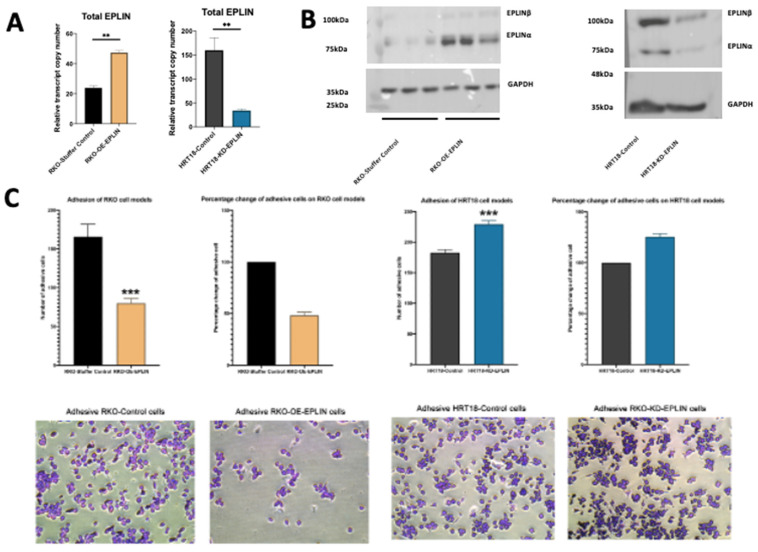

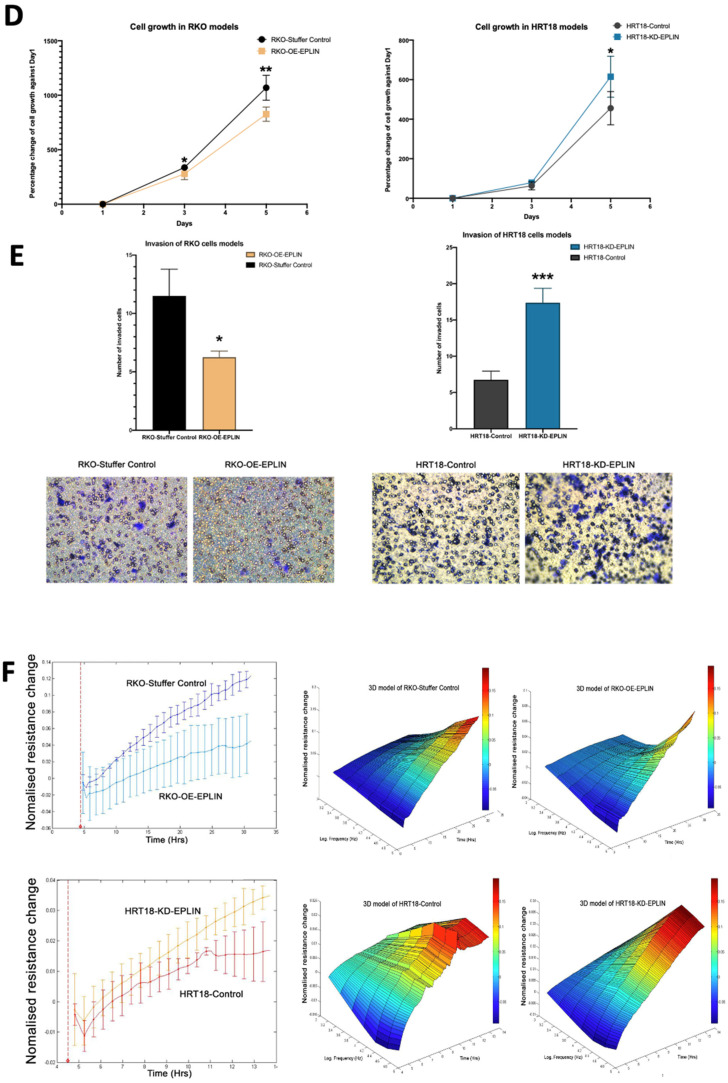
Functional implication of EPLIN on CRC cells. (**A**) Confirmation of transfection of EPLIN in RKO and HRT18 by qPCR. Data was normalised based on GAPDH, *n* = 3, data shown represents mean ± SD. (**B**) Confirmation of the EPLIN manipulation in RKO and HRT18 by Western blot. *n* = 3, representative results shown. (**C**) Matrigel adhesion assays on RKO and HRT18 cell models, *n* = 3, data shown represents mean ± SD. (**D**) MTT-growth assays on RKO and HRT18 cell models, *n* = 3, data shown represents mean ± SD. (E) Matrigel invasion assays on RKO and HRT18 cell models, *n* = 3. (**F**) ECIS migration assays on RKO and HRT18 cell models. Data was normalised by the ECIS system and represents mean ± SD. Cells were observed under ×20 magnification. * represents *p* < 0.05, ** represents *p* < 0.01, *** represents *p* < 0.001.

To further explore the potential mechanisms played by EPLIN in colorectal cancer see in clinical subjects, we chose to create In vitro cellular models. Here, we selected two established human colorectal cancer cell lines, RKO cell which is known for its rapid growth and highly invasive and motile property and HRT18, a less invasive and less motile cell lines [26,27]. The parent line of the two cells also had contrasting levels of EPLIN in that RKO had lower levels of EPLIN than HRT18 cells (Figure 2A,B). Thus, EPLIN manipulated cellular models were created by overexpressing EPLIN in RKO cells or inhibiting EPLIN via shRNA-based transfection in HRT18 cells. Verification of such models were confirmed by qPCR and Western blotting (Figure 2A,B).

Matrigel-based adhesion assay showed that the number of adherent cells that attached to the Matrigel in RKO-Stuffer Control group were significantly higher (*n* = 5, mean = 165.8) than in the RKO-OE-EPLIN group (*n* = 6, mean = 79.8) (*p* = 0.0005). The opposite effect was observed when EPLIN was inhibited in HRT18 cells. Fewer cells attached to the Matrigel in HRT18-KD-EPLIN group (*n* = 6, mean = 125.5) than in HRT18-Control group (*n* = 6, mean = 182.9) (*p* = 0.0001) (Figure 2C).

EPLIN negatively regulated cell growth. As Figure 2D demonstrates, cells in RKO-Stuffer Control group grew around 20 precent faster (*n* = 6, mean = 337.0 percent vs. Day 1) than cells in RKO-OE-EPLIN group on Day 3 (*n* = 6, mean = 279.3 percent vs. Day 1) (*p* = 0.0395). A more profound difference was observed on Day 5, when cells in RKO-Stuffer Control group grew faster (*n* = 6, mean = 1069.2 percent vs. Day 1) than cells in RKO-OE-EPLIN group (*n* = 6, mean = 826.3 percent vs. Day 1), with a significant difference of 29.4% (*p* = 0.0011). Likewise, a significant difference of growth rate on Day 5 was noted between HRT18 cellular models. Cells in HRT18-KD-EPLIN group (*n* = 6, mean = 614.9 percent vs. Day 1), grew 34.9% faster than cells in HRT18-Control group (*n* = 6, mean = 455.7 percent vs. Day 1) (*p* = 0.0153). 

In the in vitro invasion assay (Figure 2E), there was a 45.7% change in the number of cells invaded through the Matrigel in the RKO-OE-EPLIN group (*n* = 8, mean = 6.25) compared with the RKO-Stuffer Control group (*n* = 8, mean = 11.5) (*p* = 0.0424). Meanwhile, 157.4 percent more HRT18-KD- EPLIN cells invaded through the pores (*n* = 8, mean = 17.75) than HRT18-Control cells (*n* = 8, mean = 17.375) (*p* = 0.000428).

EPLIN’s effect on cellular migration was tested by employing an ECIS based wound migration assay on established cellular models (Figure 2F). RKO-Stuffer Control cells presented a slower migration than RKO-OE-EPLIN cells after wounding (Figure 2F left panel showing an example at 8000 Hz) (*n* = 6). Three-dimensional models of each group indicated the decreased migration when EPLIN was overexpressed across 7 different frequencies (1000, 2000, 4000, 8000, 16,000, 32,000 and 64,000 Hz) (Figure 2F right two panels). Similarly, when EPLIN was inhibited, HRT18 cells displayed a faster migration than control group (8000 Hz) (*n* = 6). Three-dimensional models demonstrated a clearer indication at 7 different frequencies, especially after the 11 h time point, HRT18-KD-EPLIN group presented higher normalised resistance value compared with the HRT-Control group. 

Therefore, our in vitro cellular assays demonstrated that EPLIN negatively regulated the growth, adhesion, migration and invasion in CRC cells.

### 2.4. Her2 and HSP60, Potential Novel Partners of EPLIN, and Their Clinical Implication on CRC Patients

A Kinexus^TM^ antibody-based KAM-880 protein microarray (Kinexus Bioinformatics Ltd., Vancouver, BC, Canada) was employed on two pairs of CRC patients’ protein samples from paired normal colon mucosa tissues and tumour tissues, to further explore potential interacting partners of EPLIN in CRC. As shown in Table 3, a number of protein kinases were found to differentially interacted with EPLIN. Mitogen- Activated Protein (MAP) kinase family members and their downstream kinases were also observed to be aberrant, namely MAPK9 (JNK2), MAPK7 (ERK5), MAPK8 (JNK1), MAPK3 (ERK1) and MAPK Interacting Serine/Threonine Kinase 2 (MKNK2). Intensity of protein kinases that relate to MAPK/ERK pathways were observed to be dysregulated, such as B- Raf, PDGFRa and PDGFRb. ERK1 and STAT3, two elements that EPLIN has been implicated to interact with to regulate the EMT process [18,20], were also observed to be aberrant. Additionally, PKA, a known interacting partner of EPLIN, which is involve in apical elimination jointly [28] was also noted.

**Figure 3:**
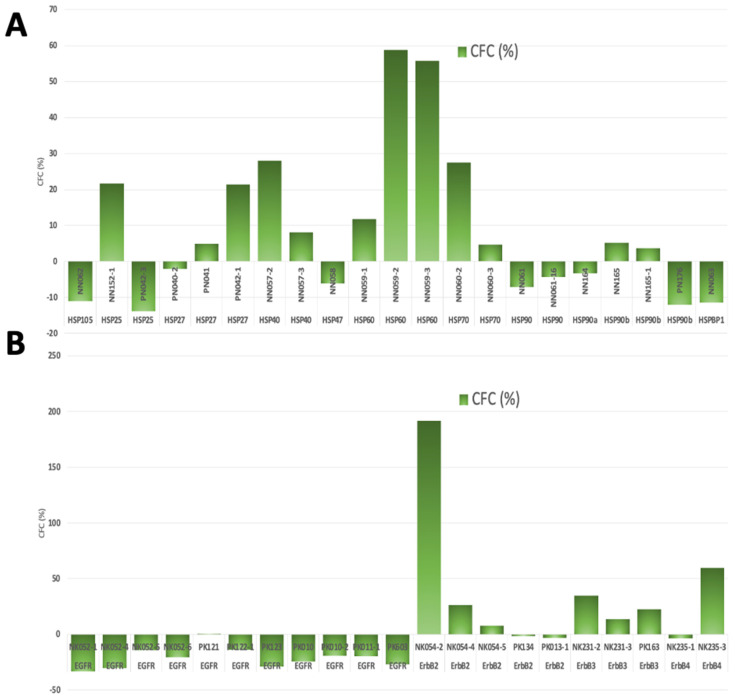

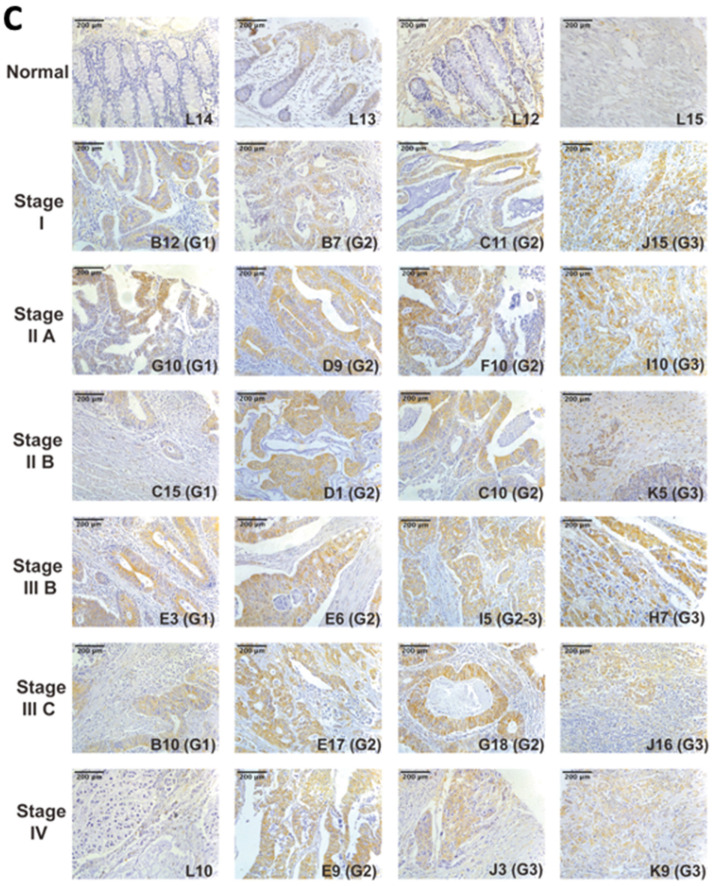
Analysis of Kinexus^TM^ protein microarray and IHC assay of HSP60 on CRC TMA. (**A**) Analysis of CFC (%) of HSP family due to the downregulation of EPLIN. (**B**) Analysis of CFC (%) of EGFR family due to the downregulation of EPLIN. Data was obtained from the result report from the Kinexus protein microarrays. (**C**) IHC staining of HSP60 in the colorectal cancer TMA (CO2161a).

The protein complexes that were of significant interest to this study were the Heat Shock Protein (HSP) and Epidermal Growth Factor Receptor (EGFR) family members which were noted to be aberrant between the pairs of normal and tumour colon tissues (Figure 3A,B). As Figure 3A demonstrated, all three antibodies in the protein array platform that targeted HSP60 showed the trend of upregulation (NN059-1&2&3). In which NN059-2 and NN059-3 showed a more dramatic changes (NN059-2: CFC%: 59; NN059-3: CFC%: 56) and were labelled as priority potential partners. Similarly, signal intensity of all four members of EGFR family were tested (Figure 3B). The highlight of the findings was that three different antibodies, pan-specific for Her2 (NK054-2, NK054-4 and NK054-5), showed a trend of enhanced signal intensity between normal samples and tumour samples. NK054-2 also demonstrated a 192% CFC change and was identified as a priority target. Two phosphorylation sites of Her2 were also tested (PK134: T686 and PK013-1: Y1248), no outstanding difference between normal and tumour samples was noted. Hence, we showed signalling events and interacting partners that may be involve in EPLIN’s network. Her2 and HSP60 were chosen to process further investigation.

The transcript expression profile of HSP60 and Her2 were accessed by revisiting our clinical colorectal cohort (Table 4 and Table 5). As demonstrated in Table 4, median transcript expression of HSP60 (*n* = 94, median = 0.45) was significantly upregulated in tumour samples when compared with normal tissues (*n* = 80, median = 0.05, *p* = 0.0097). Additionally, transcript expression of HSP60 was found to be related to TNM stages (*p* = 0.045) and T stages (*p* = 0.033). When samples were divided based on TNM stages, median transcript expression in TNM1 group (*n* = 9, median = 53.9) was observed to be higher than its expression in TNM2 (*n* = 30, median = 0.1), TNM3 (*n* = 26, median = 0.1) and TNM4 (*n* = 6, median = 32.9) groups. Similarly, the T1 group was found to have higher median transcript expression of HSP60 (*n* = 2, median = 152) than in T2 (*n* = 10, median = 54.3), T3 (*n* = 40, median = 0.09) and T4 (*n* = 18, median = 0.4). No statistical significance was noted in comparison of other pathological information. 

Additionally, IHC analysis was performed on the CRC TMA samples (CO2161a) (US Biomax, supplied by Insight Biotechnologies, Middlesex, UK) to assess HSP60 at the protein level (Table 6 and Figure 3C). All normal tissues were rated negative to weak for HSP60 staining (*n* = 8), while 83 of a total of 175 adenocarcinoma tissues were scored as moderate to strong staining for HSP60 (Table 6). Intriguingly, 26 of a total of 30 mucinous adenocarcinoma tissues showed negative to weak staining and reached statistical significance when compared to the adenocarcinoma group (*p* < 0.01). As demonstrated in Figure 3C HSP60 staining was mainly distributed in the cytoplasm of cells and was generally stronger in tumour samples than in normal ones (B12, stage I, well differentiated & J15, stage I, poorly differentiated vs. L14, normal tissue). The staining intensity of HSP60 was also observed to be related to stages. Moderate to strong staining of HSP60 accounted for 38.9% in the Stage-1 group, 40.9% in the Stage-2 group and 45.7% in the Stage-3 group. The difference is nonetheless not significant (*p* > 0.05). As Figure 3C shows, HSP60′s intensity was generally stronger in D9 (Stage2A), D1 (Stage2B), E6 (Stage3B) and E17 (Stage3C) than in B12 (Stage1) and B7 (Stage1). To our surprise, 3 of 4 tissues in the Stage4 group showed negative to weak staining of HSP60. HSP60 staining was otherwise not to be related to the differentiation of colorectal cancer (Table 6). 

We also analysed the transcript expression profile of Her-2 (Table 5). Her-2 median transcript expression was found to be higher in tumours samples than in normal tissues (0.4103 vs. 0.0043, *p* < 0.001). Median transcript expression of Her-2 was positively correlated with T stages, but no statistical significance was noted (*p* = 0.571). Likewise, its median transcript expression was higher in patients with distant metastatic lesions (*n* = 50, median = 0.413) compared to those without (*n* = 19, median = 1.123), and in patients with cancer related incidence, compared with those without. These differences were not statistically significant, potentially owing to the limitation of the sample numbers. Collectively, we demonstrated that CRC tumour samples expressed higher HSP60 at transcript and protein levels than normal ones. While Her2’s expression was upregulated in CRC tumour samples at transcript level. 

### 2.5. Implication of Her2, HSP60 and EPLIN on CRC Patient Prognosis

**Figure 4:**
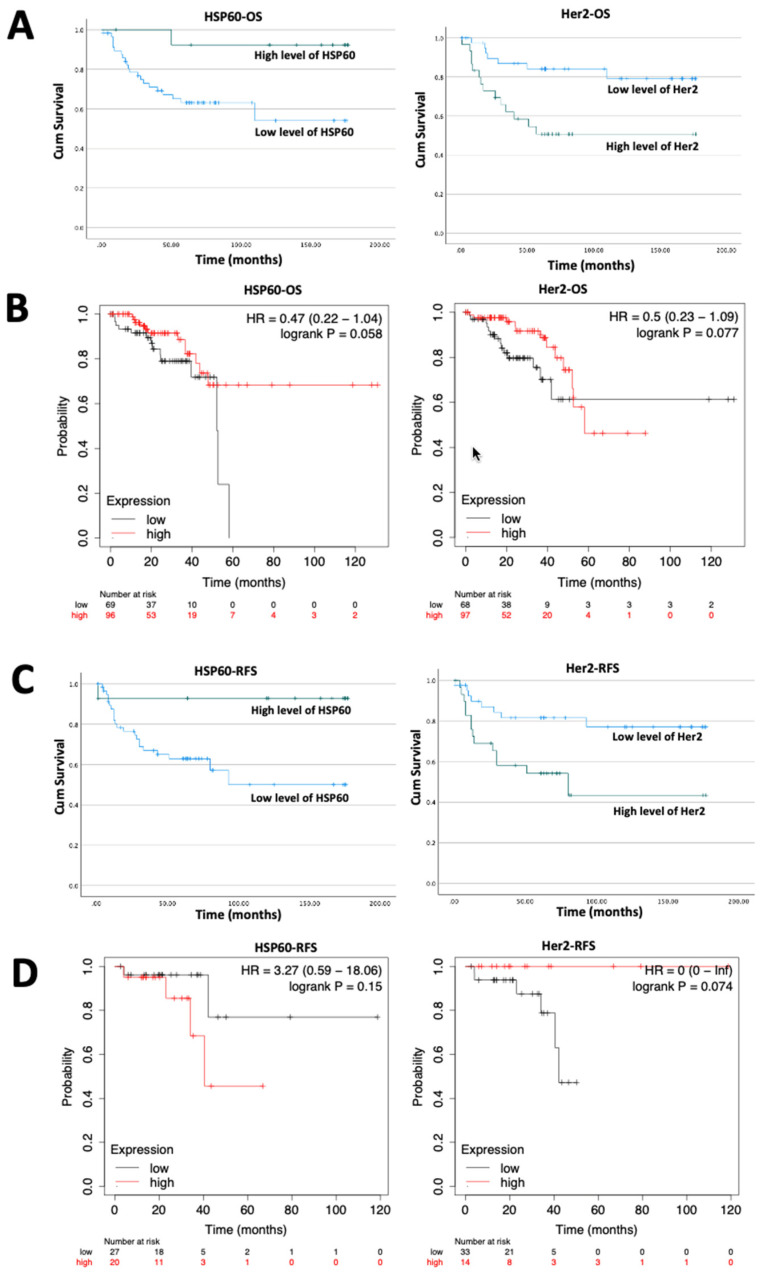

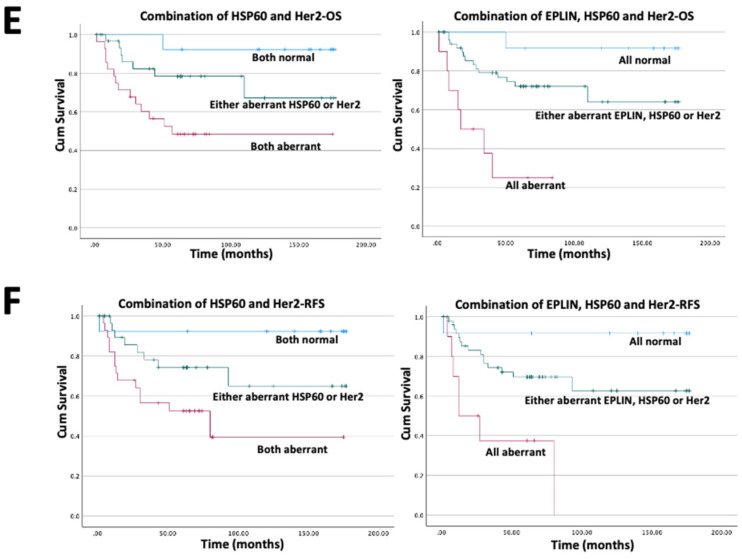
Implication of Her2, HSP60 and EPLIN on CRC patient’s survival. (**A**) Implication of HSP60 and Her2 on patient’s OS in the clinical cohort. (**B**) Survival curves of OS in rectum adenocarcinoma by probing HSP60 and Her2 on the KM plotter platform. (**C**) Implication of HSP60 and Her2 on patient’s RFS in the clinical cohort. (**D**) Survival curves of RFS in rectum adenocarcinoma by probing HSP60 and Her2 on the KM plotter platform. (**E**) Implication of the combination of HSP60 and Her2/EPLIN on patient’s OS in the clinical cohort. (**F**) Implication of the combination of HSP60 and Her2/EPLIN on patient’s RFS in the clinical cohort.

After accessing the transcript expression profiles of Her2 and HSP60 in our CRC clinical cohort, their relationships with patient’s survival were explored by analysing the local Cardiff cohort and KM plotter dataset (Figure 4). As demonstrated in Figure 4A, patients with high level of HSP60 (*n* = 14, mean = 167.231 months) had a significantly better OS than those with lower level of HSP60 (*n* = 59, mean = 113.8 months; *p* = 0.025). While patients with high level of Her2 (*n* = 40, mean = 101.3 months) had a significantly worse OS than those with low level of Her2 (*n* = 19, mean = 149.4 months; *p* = 0.003). Exploration of the rectum adenocarcinoma dataset on KM plotter revealed the similar result (Figure 4B). Patients with high level of HSP60 tended to have a better OS than those with low level of HSP60, a more dramatic difference was observed after a 60 month-time point, demonstrating near significance (*p* = 0.058). Likewise, patients with high levels of Her2 tended to have a worse OS than those with low level of Her2 after 60 months, though again, no statistical significance was noted. 

Implication on RFS was also investigated. As shown in Figure 4C, CRC patients in our clinical cohort who had high level of HSP60 (*n* = 14) were observed to have a significantly better RFS than those with low level of HSP60 (*n* = 59) (mean: 164.4 months vs. 106.4 months; *p* = 0.022). Patients with high level of Her2 (*n* = 30) had a significantly worse RFS than those with low level of Her2 (*n* = 42) (mean: 93.4 months vs. 143.6 months; *p* = 00008). Similar effects were observed in rectum adenocarcinoma patients following analysis using KM plotter (Figure 4D) in which patients with high level of HSP60 tended to have a better RFS than those with low level of HSP60. Patients who expressed high level of Her2 tended to have a worse RFS than those who expressed low level of Her2. However, neither reached statistical significance (HSP60: *p* = 0.075; Her2: *p* = 0.15). Hence, CRC patients with high level of HSP60 were indicated to have a better OS and RFS. These findings from the KM plotter dataset seem to be conflict with HSP60′s expression profile between CRC tumour samples and normal samples. Meanwhile, high levels of Her2 was indicated to affect CRC patient’s OS and RFS.

With the findings that HSP60 and Her-2 interacted with EPLIN, the power of an integrated combination of these three partner proteins was considered for further investigation. As Figure 4E (left) and Figure 4F (left) showed, patients were divided into three groups based on expression of HSP60 and Her2: patients with no aberrant expression of HSP60 or Her2 (*n* = 13), patients with either abnormal expression of HSP60 or Her2 (*n* = 33) and patients with abnormal expression of both HSP60 and Her2 (*n* = 28). Patients with either abnormal expression of HSP60 or Her2 (OS: mean = 136.3 months; RFS: mean = 129.3 months) had a worse OS and RFS than patients with no aberrant expression of HSP60 or Her2 (OS: mean = 167.2 months; RFS: mean = 163.5 months). While patients with both abnormal expression of HSP60 and Her2 had the worst OS and RFS among three groups (OS: mean = 96.9 months; RFS: mean = 87.7 months) (OS: *p* = 0.004; RFS: *p* = 0.009). More interestingly, patients were divided into three groups based on their expression of EPLIN, HSP60 and Her2, namely patients without any aberrant expression of such molecules (*n* = 12), patients with either abnormal expression of EPLIN, HSP60 or Her2 (*n* = 52) and patients with abnormal expression of EPLIN, HSP60 and Her2 (*n* = 10). It was very interesting to note that patients who expressed abnormal expression of EPLIN, HSP60 and Her2 (OS: mean = 35.1 months; RFS: 37.7 months) had the worst OS and RFS compared with patients expressing either abnormal expression of them (OS: 129.4 months; RFS: 124.2 months) and patients who did not expressed any aberrant expression of them (OS: 166.4 months; RFS: 162.3 months) and this trend was found to be significant (both *p* < 0.001). Furthermore, the power of the combination of EPLIN, HSP60 and Her2 was examined by performing a multivariate analysis using Cox regression model (Table 7 and Table 8). As shown in Table 7, none of the pathological nor clinical indicators tested was shown to have a significant value, but the combination of EPLIN, HSP60 and Her2 presented as a significant predictor for patient’s OS (Hazard Ratio: 5.461, *p* = 0.024). Similarly, as Table 8 demonstrated, both Ducks stage and TNM stage were identified as significant predictors for RFS of CRC patients (*p* = 0.021 and *p* = 0.04, respectively). The combination of EPLIN, HSP60 and Her2 was also indicated to be a significant predictor for RFS as well (Hazard Ratio: 2.929, *p* = 0.049). 

### 2.6. Regulatory Relationship between EPLIN, HSP60 and Her2

We identified Her2 and HSP60 could be two novel potential EPLIN interacting partners by carrying out Kinexus^TM^ antibody-based protein microarray on protein samples from patients. In order to verify such interaction, immunoprecipitation was carried out on protein samples from CRC cell lines, to precipitate EPLIN/HSP60/Her2 and probe with antibodies of the other respective partners. However, no direct protein–protein interaction was detected, indicating that the partners may interested act indirectly. We went on to analyse the relationship between the three molecules in the cohort (Table 9). EPLIN transcript positively correlated with HSP60 (r = 0.488, *p* < 0.01), but negatively correlated with Her3 (r = 0.272, *p* = 0.018). Meanwhile, HSP60 also had significant positive correlations with Her1 (r = 0.406, *p* < 0.01) and Her4 (r = 0.248, *p* = 0.017). There was no significant correlation between Her2 and HSP60/EPLIN.

**Figure 5:**
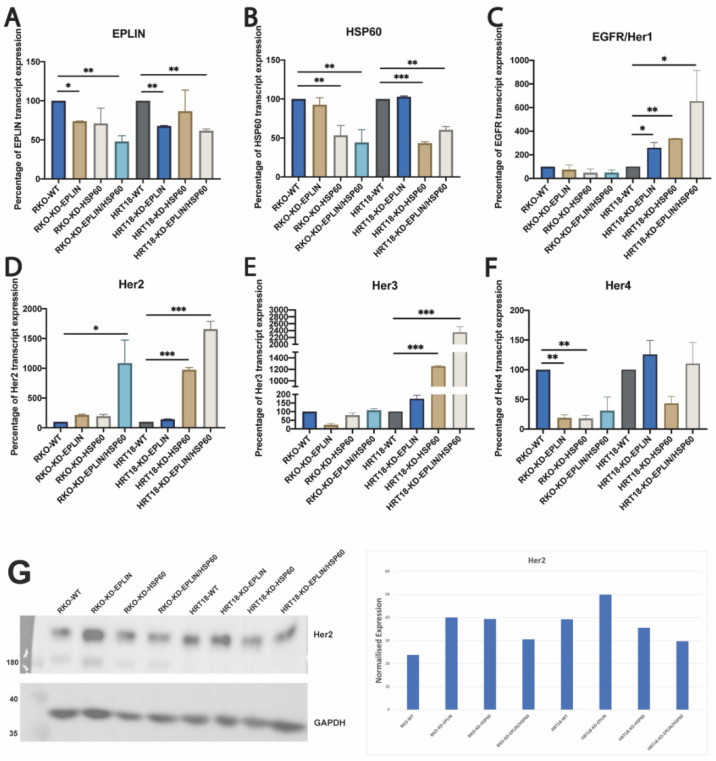
Regulatory relationship between EPLIN, HSP60 and EGFR family, investigated by quantitative PCR in cell models. (**A**) Expression of EPLIN transcript in RKO and HRT18 cell models. (**B**) Expression of HSP60 transcript in RKO and HRT18 cell models. (**C**) EGFR transcript expression in cell models. (**D**) Her2 transcript expression in cell models. (**E**) Her3 transcript expression in cell models. (**F**) Her4 transcript expression in cell models. Expression data was normalised by GAPDH. (**G**) Confirmation of change of Her2 protein following EPLIN and HSP60 modification in HRT18 and RKO cells. Shown are protein electrophoresis gel (left) and the quantified protein band normalised to GAPDH (right). Data represents mean ± SD, *n* = 3, * represents *p* < 0.05, ** represents *p* < 0.01, *** represents *p* < 0.001.

We further created cell models by inhibiting EPLIN/HSP60 alone or together in CRC cell lines to investigate if EPLIN and HSP60 exerted regulatory influence of each other (Figure 5). After inhibiting HSP60 alone in RKO and HRT18 (RKO/HRT18-KD-HSP60), no significant difference of EPLIN was noted due to its manipulation (Figure 5A). Similarly, inhibition of EPLIN alone in both cell lines (RKO/HRT18-KD-EPLIN) did not induce changes of HSP60 (Figure 5B). Interestingly, both EPLIN and HSP60 was indicated to regulate EGFR family members (Figure 5C). When EPLIN or HSP60 was inhibited alone in HRT18, transcript expression of EGFR was upregulated significantly when compared with the WT group (HRT18-WT: mean = 1.59 ± 0.47; HRT18-KD-EPLIN: mean = 4.11 ± 1.25, *p* = 0.031; HRT18-KD-HSP60: mean = 5.37 ± 0.01, *p* = 0.002). A more dramatic upregulation of EGFR was noted when EPLIN and HSP60 were supressed together in HRT18 (HRT18-KD-EPLIN/HSP60: mean = 9.85 ± 4.24, *p* = 0.028). Such regulation was not observed in RKO cellular models. Regarding Her2 (Figure 5D), inhibition of EPLIN or HSP60 alone in RKO led to an upregulated trend of transcript Her2 levels (*p* > 0.05). However, dual inhibition of EPLIN and HSP60 in RKO resulted in a significant upregulation of Her2 transcript expression (RKO-WT: mean = 1.09 ± 0.62; RKO-KD-EPLIN/HSP60: mean = 11.79 ± 4.23, *p* = 0.043). 

Her2 transcript level in HRT18 cells was upregulated following inhibition of HSP60 alone or with EPLIN/HSP60 when compared with the WT group (HRT18-WT: mean = 20.27 ± 8.43; HRT18-KD-HSP60: mean = 197.34 ± 7.82, *p* < 0.001; HRT18-KD-EPLIN/HSP60: mean = 336.06 ± 26.47, *p* < 0.001). No statistical significant difference in Her2 transcript level was noted after inhibiting EPLIN in HRT18 cells. As Figure 5E demonstrated, transcript level of Her3 was also upregulated when HSP60 was supressed with or without EPLIN in HRT18 cells (HRT18-WT: mean = 21.09 ± 7.62; HRT18-KD-HSP60: mean = 264.41 ± 5.22, *p* < 0.001; HRT18-KD-EPLIN/HSP60: mean = 494.96 ± 61.43, *p* < 0.001). An upregulated trend was also noted after inhibiting EPLIN alone although this did not reach significance (*p* = 0.067). Additionally, transcript levels of Her4 were found to be downregulated in RKO after manipulating EPLIN and HSP60 alone or together (Figure 5F). Inhibition of EPLIN and HSP60 alone in RKO, resulted in a significant downregulation of Her4 compared to WT group (RKO-WT: mean = 8.70 ± 1.001; RKO-KD-EPLIN: mean = 1.64 ± 0.79, *p* = 0.003; RKO-KD-HSP60: mean = 1.55 ± 0.79, *p* = 0.003). In conclusion, our results suggested that EPLIN and HSP60 regulate Her2 and Her4 in RKO cells at the transcript level, while they regulate EGFR and Her3 in HRT18 at the transcript level. The change of Her2 following EPLIN knockdown was also noted at protein levels as shown in Figure 5G.

### 2.7. EPLIN and HSP60 Have Potential to Regulate Chemo- and Targeted-Therapeutic Resistances

**Figure 6:**
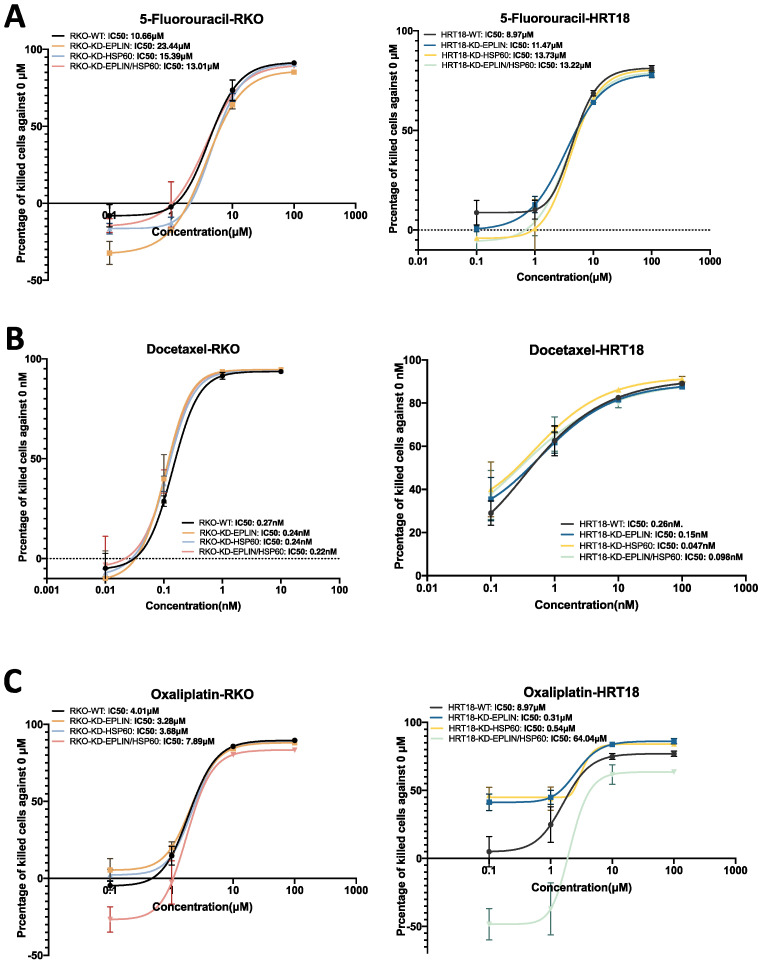
Implication of EPLIN and HSP60 on CRC cell’s response to chemotherapeutic agents. (**A**). Cytotoxicity assays on 5-FU (0–1000 μM) in RKO and HRT18 cell models. (**B**). Cytotoxicity assays on Docetaxel (0–100 nM) in RKO and HRT18 cell models. (**C**). Cytotoxicity assays on Oxaliplatin (0–1000 μM) in RKO and HRT18 cell models.

**Figure 7:**
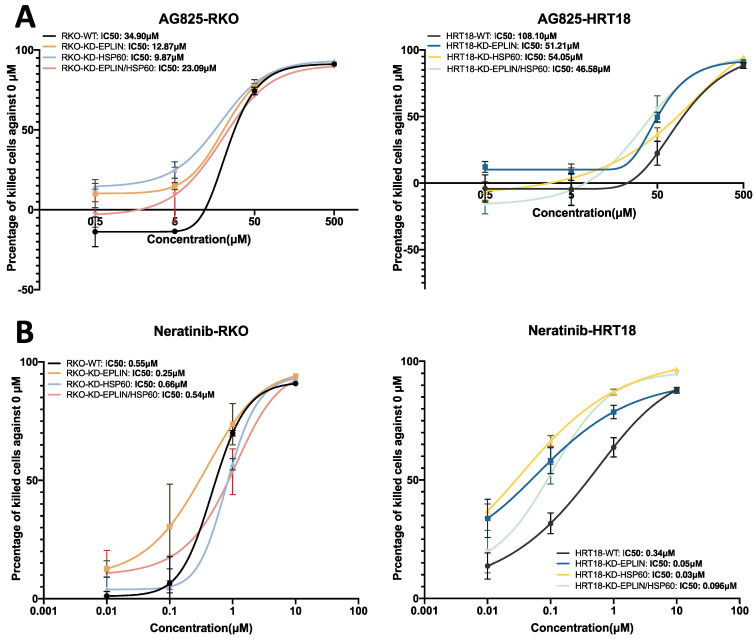
Implication of EPLIN and HSP60 on CRC cell’s response to AG825 and Neratinib. (**A**) Cytotoxicity assays on AG835 (0–500 μM) in RKO and HRT18 cell models. (**B**) Cytotoxicity assays on Neratinib in RKO and HRT18 cell models (0–100 μM).

EPLIN has been implicated to play a role in drug resistance in prostate cancer [12] and gastric cancer [29]. Likewise, HSP60 has also been implicated in mediating drug resistance in ovarian cancer [30] and colorectal cancer [31]. In light of this, we further investigated the possible role played by EPLIN and HSP60 in the responsiveness of colorectal cancer cells to chemotherapeutic and EGFR/Her2 targeted therapeutic agents. RKO and HRT18 cellular models displaying manipulated expression of EPLIN/HSP60 were subject to cytotoxicity assays with chemotherapeutic agents (5-Fluorouracil, Docetaxel and Oxaliplatin), Her2 inhibitor (AG825) and EGFR/Her2 targeted therapeutic agents (Neratinib) (Figure 6 and Figure 7).

As Figure 6A and Table 10 show, 5-Fluorouracil (5-FU) had an IC50 of 10.66μM in the RKO-WT group, rising to 23.44 μM when EPLIN was inhibited. Less significant changes in IC50s were seen when HSP60 was inhibited with or without EPLIN in RKO (RKO-KD-HSP60 = 15.39 μM, RKO-KD- EPLIN/HSP60 = 13.01 μM) compared to the WT group. Similar results were observed in the HRT18 models, though at a lesser degree in that inhibition of EPLIN in HRT18 led to decreased IC50 when compared to HRT18-WT group (11.47 μM vs. 8.97 μM). Similar trends were also observed when HSP60 was inhibited alone or with EPLIN in HRT18 (IC50: HRT18-KD-HSP60 = 13.73 μM, HRT18-KD- EPLIN/HSP60 = 13.22 μM). Hence, EPLIN was suggested to affect cellular response to 5-Fluorouracil.

We did not observe a significant difference in the response to Docetaxel between each RKO cellular model (Figure 6B Left). Interestingly, inhibition of HSP60 in HRT18 led to an increased IC50 compared to WT group (0.047 nM vs. 0.15 nM) (Figure 6B right). Although inhibition of EPLIN did not seem to affect cell’s response to Docetaxel in HRT18, knocking down both molecules together in HRT18 also increased the IC50 compared to WT group (0.098 nM vs. 0.15 nM). Thus, inhibition of HSP60 in HRT18 increased the responsive efficiency to docetaxel. This effect seems to be interfered with by suppressing EPLIN in HRT18.

Inhibition of EPLIN or HSP60 alone in RKO did not result in obvious changes in the IC50s of Oxaliplatin compared to the WT group (Figure 6C Left), while knocking down both molecules together in the cell lines seemed to reduce the cell’s response to Oxaliplatin (IC50s: RKO-WT = 4.01 μM, RKO-KD-EPLIN/HSP60 = 7.89). In the HRT18 cellular models (Figure 6C Right), inhibition of EPLIN or HSP60 alone increased the cell’s response (IC50s: HRT18-WT = 4.51 μM, HRT18- KD-EPLIN = 0.31 μM, HRT18-KD-HSP60 = 0.54 μM). Interestingly, HRT18 became more resistance to Oxaliplatin when both molecules were inhibited together (IC50: HRT18-KD-EPLIN/HSP60 = 64.04 μM). Hence, inhibition of RKO or HSP60 alone seem to increase the cell’s response to Oxaliplatin, while the responsive efficiency was supressed when both molecules were inhibited together.

Finally, we tested the response of cells to two of the Her2 inhibitors, namelyAG825, a selective Her2 inhibitor and Neratinib a broad spectrum Her2 inhibitor used in cancer treatment (Figure 7A,B, Table 10). Following knock down of EPLIN, HSP60 or EPLIN/HSP60, both HRT18 and RKO showed an increased sensitivity to both inhibitors with decreased IC50s. The RKO cells responded in a similar but much weaker pattern. 

Collectively, this indicates that EPLIN and HSP60 have the potential to regulate colorectal cancer cell’s response to both chemotherapeutic and EGFR/Her2 targeted therapeutic agents.

## 3. Discussion

EPLIN has been implicated as a tumour suppressor and is associated with patient’s clinical outcomes as well as drug resistances in some cancer types. In the present study, we have shown that EPLIN is also an important regulator in colorectal cancer. Downregulation of EPLIN was found in colorectal cancer at transcript and protein levels and this downregulation was also related to poor OS and RFS in colorectal cancer patients. In vitro, we demonstrated that genetic modification of EPLIN expression rendered cells with less aggressive phenotypes, impacting growth, adhesion, migration and invasion negatively. These properties of EPLIN on colorectal cancer cells thus indicates itself as an important player in influencing some of the hallmarks of cancer, namely invasion and metastasis [32,33]. These findings collectively suggest that in colorectal cancer, EPLIN behaves as a potential tumour suppressive molecule, as suggested in a number of other cancer types [11,12,13,14,15,17,18,34].

Apart from emphasizing EPLIN’s impact on clinical outcomes and cellular functions, we also search for potential mechanistic or interacting partners. Our search for potential EPLIN partners in colon cancer cells utilising Kinexus^TM^ antibody-based protein microarray identified HSP60 and Her2 are as potential interacting partners, together with a few others related to MAPK signalling pathway. By following this path, we did not observe direct protein–protein interaction between EPLIN, HSP60 and Her2. This could be due to the limitation of in vitro study based on epithelial colorectal cancer cell lines. Proteins that were applied to the protein microarray contains not only epithelial cells, but also elements from microenvironment, such as stomal cells, stem cells, etc. Thus, this close interaction might exist in other elements but not in epithelial cells. After manipulating the expressions of EPLIN and HSP60 in RKO and HRT18 cell lines, the regulatory relationship between EPLIN, HSP60 and EGFR family members were investigated. Interestingly, in both cell lines, inhibition of EPLIN and HSP60 together resulted in a marked upregulation of transcript level of Her2. This interesting finding was supported by our analysis on clinical cohort, in that HSP60 expression was found to be upregulated in Her2 positive breast cancer and such regulation was predicted to be affected by MAPK signalling [35]. EGFR and Her3 were only found to be regulated in HRT18 cellular models, while Her4 was found to be regulated in RKO cellular models. Such results might be due to the different genetic profiles between RKO and HRT18. For example, Her4 was mutated in HRT18 but not in RKO. 

We also assessed transcript profiles of HSP60 and Her2 in clinical colorectal cancer cohorts and highlighted that high levels of HSP60 and Her2 were observed in tumour tissues compared to normal ones. However, based on our analysis of a clinical cohort and KM plotter, patients with high level of HSP60 was found to have better OS and RFS. These findings seem in conflict to our observation on transcript profile. Vocka et al. reported that high serum level of HSP60 was related to worse OS in colorectal cancer patients [36]. Hence, a larger cohort is needed for better understanding of the clinical impact of HSP60. However, the significant finding that the combination of EPLIN, HSP60 and Her2 expression presents a significant independent prognostic indicator for the clinical outcome of the patients strongly argues that this interacting partner group has a value that need to be further explored in this cancer type and potentially in other types of cancers.

Cytotoxicity assays revealed EPLIN and HSP60 might have the potential to affect chemotherapeutic and targeted therapeutic resistances. Inhibition of both molecules in RKO and HRT18 resulted in less sensitive response to 5-FU. While more sensitive cell’s response to Docetaxel was found in HRT18 when both molecules were suppressed. Interestingly, inhibition of either of the molecules alone in HRT18 increase cell’s response to Oxaliplatin, but an opposite effect was observed when both were inhibited together. Therefore, we showed the potential of EPLIN and HSP60 in regulating cell’s response to chemotherapeutic agents. Inhibition of EPLIN and HSP60 alone or together in our cellular models resulted in more sensitive response to an HER2 inhibitor and Neratinib. This might be related to the upregulation of Her2 caused by inhibiting EPLIN and HSP60 and argues a novel direction for identifying patients with colorectal cancer who have a favourable pattern of expression of the molecular pair and may be more sensitive to target therapies to Her family. 

Since its first discovery more than two decades ago [10], there have been sustained efforts in not only determining its role in cancer progression and metastasis, but also the potential mechanism(s) played by EPLIN in cancer cells. As it has been recently summarised [37], a small number of potential leads have been reported to be potential partners of EPLIN including p53, caveolin-1, plectin, paxillin and FAK (focal adhesion kinase). These potential partners are known players in cell adhesion and migration and cell growth. These early findings together with the discovery of the present study contribute to the unveiling of how EPLIN interplays in the complex networking within the ‘blackbox’ of the cells and its networking. However, it is also clear that there is a long way to go to fully clarify the signalling events up and down stream of EPLIN, an exciting prospect to further expand this area, together with the understanding of its role of a broad sense of clinical cancers. 

In conclusion, EPLIN appears to act as a tumour suppressor in clinical colorectal cancer and regulates cellular growth, invasion, migration and adhesion negatively in colorectal cancer cells. Her2, HSP60 and their related signalling events (i.e.,MAPK) are potential novel interacting partners of EPLIN. Aberrant expression of EPLIN, HSP60 and Her2 is identified as an independent predictor for OS and RFS. EPLIN and HSP60 have the potential to regulate cell’s response to chemotherapeutic agents. While inhibition of both molecules leads to upregulation of Her2 and more sensitive cell’s response to Her2 inhibitor and Neratinib. These findings warrant more intensive studies on EPLIN both in clinical and therapeutics in colorectal cancer. 

## 4. Materials and Methods

### 4.1. Collection and Processing of Colorectal Cancer Tissues 

A clinical cohort of colorectal cancer containing 94 colorectal cancer tissues from the patients. Of these 94 samples, we were able to obtain 80 normal matched colorectal cancer samples from the same patients. The normal colon tissues were obtained from the same patients with at least 10 cm away from tumour margins. Both tumour and normal tissues were used in the present study. Tissues were collected in the University Hospital of Wales after surgery and consenting the patients. Tissue samples were examined by a consultant pathologist and stored at −80 °C until use. Pathological information was obtained via hospital clinical portal system and CANISC data. Tissues were used under ethical approval by East Wales Local Research Ethics Committee (reference number SJT/C617/08). 

### 4.2. Cell Culture

Two colorectal cancer cell lines were used for this study, RKO (CRL-2577, poorly differentiated colorectal carcinoma cell line derived from colon and with no known P53 mutation) and HRT18 (ECACC 86040306, an epithelial adherent cell line derived from large intestine. Both cell lines were purchased from American Type Culture Collection (ATCC, Rockville, MD, USA) (LGC Standard, ATCC UK agent). Cells were cultured at low passages at 37 °C with 95% humidity and 5% CO_2_ in either Dulbecco’s Modified Eagle’s medium (DMEM) or RPMI-1640 medium with L-glutamine and sodium, which were supplement with 10% heat inactivated foetal calf serum (FCS) and 1% antibiotics mixture (Sigma-Aldrich, Pooled, Dorset, UK).

### 4.3. RNA Extraction and Reverse Transcription

TRI Reagent Kit (Sigma-Aldrich, Poole, Dorset, UK) was used to extract RNA from tissues samples and cells from colorectal cancer cell lines following manufacture’s instruction. After isolation, RNA samples were quantified to 500 ng/μL and used to performed reverse transcription in a Simpliamp thermocycler (Fisher Scientific UK Leicestershire, UK), using a GoScript^TM^ Reverse Transcription System Kit (Promega Corporation, Madison, WI, USA). cDNA samples were then stored at −20 °C until required.

### 4.4. Real Time Quantitative PCR (qPCR)

qPCR was carried out to assess transcript expression of genes of interest. Amplifilour Uniprimer^TM^ Universal system (Intergen company, New York, USA) was utilised for qPCR [29]. In brief, each reaction was made up with 5 μL precision FAST2x qPCR Master Mix (PrimerDesign, Southampton, UK), 0.3 μL forward primers (EPLIN: AAGCAAAAATGAAAACGAAG; GAPDH: AAGGTCATCCATGACAACTT; Her1: GACCTCCATGCCTTTGAGAA; Her2: CCTCCTCGCCCTCTTG; Her3: CCCCACACCAAGTATCAGTA; Her4: CTGCTGAGTTTTCAAGGATG; HSP60: TGTAGACCTTTTAGCCGATG), 0.3 μL reverse primer with z sequence whose concentration was 1/10 of forward primer (EPLIN: ACTGAACCTGACCGTACAGACACCCACCTTAGCAATAG; GAPDH: ACTGAACCTGACCGTACAGCCATCCACAGTCTTCTG; Her1: ACTGAACCTGACCGTACAGCACAAATTTTTGTTTCCTGA; Her2: ACTGAACCTGACCGTACACATGTCCAGGTGGGTCT; Her3: ACTGAACCTGACCGTACAACACAGGATGTTTGATCCAC; Her4: ACTGAACCTGACCGTACAAACTTGCTGTCATTTGGACT; HSP60: ACTGAACCTGACCGTACAACAGTCACACCATCTTTTGT) (Sigma-Aldrich Co, Poole, Dorset, UK), 0.3 μL uniprimer and 4.1 μL cDNA samples. In addition to the test cDNA samples, a set of known transcript copy number samples (ranging from 10^1^ to 10^8^) was also run as standard relative copy number of cDNA samples were calculated based on this standard and normalised to the housekeeping control, GAPDH.

### 4.5. Preparation of Protein Samples 

Protein samples were collected from colorectal cancer patients or from cell lines. In brief, colorectal cancer cell lines were harvested with PBS once they reached desired confluences (<90%). After centrifuging, supernatant was discarded and pellet was resuspended with home-made modified RIPA lysis buffer. Samples were put on a rotational platform and incubated overnight at 4 °C before being centrifuged at 4 °C to form pellets. Pellets was then discarded and samples processed and quantified. The concentration of samples was tested using a BioRadDC Protein Assay (BioRad Laboratories, Hertfordshire, UK) in accordance with the manufacturer’s instructions. Samples were quantified to at least 2 μg/μL with lysis buffer and were stored at −20 °C before use. 

### 4.6. Western Blotting 

Western blotting was performed to detect expression of protein samples. Briefly, protein samples were loaded on a Sodium dodecyl sulfate-polyacrylamide (SDS-PAGE) gel. The gel was subject to electrophoresis to separate proteins based on mass at 120 V, 50 W and 50 mA until sufficient separation was obtained. The gel was then transferred to an Immobilon-P PVDF membrane (Merck Millipore, Hertfordshire, UK) to perform semi-dry transfer via a semi-dry blotter at 15 V, 500 mA, 20 W for 50 min. Immunoblotting was then carried out as follows: The membrane was blocked with 5% milk mixture which was diluted in tris buffered saline (TBS) (Sigma-Aldrich Co, Poole, Dorset, UK) with 0.1% tween-20 (Melford Laboratories Ltd., Suffolk, UK) for an hour before incubating with desired primary antibody (anti-EPLIN: monoclonal, mouse, dilution 1:500; sc-136339; anti-GAPDH: monoclonal, mouse, dilution 1:1000, sc-32233; anti-Her2, mouse monoclonal, SC-33684 dilution 1:500; purchased from Santa Cruz Biotechnology, Inc. Dallas, Texas, USA) (diluted with 2.5% mixture) overnight at 4 °C. The membrane was washed in 2.5% milk mixture three times for 15 min at room temperature then was incubated with secondary antibody, mouse (whole molecule) IgG peroxidise conjugate (Sigma-Aldrich Co, Poole, Dorset, UK), which was diluted 1000 times in 2.5 milk mixture, for an hour at room temperature. After incubation, membrane was washed with TBS-T and TBS twice for 10 min. EZ-ECL solution (equal parts of solution A mix with solution B) (Geneflow Ltd., Litchfield, UK) was used to incubate with membrane in the dark before capturing images on a G-BOX (Syngene, Cambridge, UK) detection system.

### 4.7. Immunohistochemical Staining and Analysis

Immunohistochemical staining was performed on the tissue microarray (TMA) slides from colorectal cancer (https://www.biomax.us/tissue-arrays/Colon/CO2161a, accessed on 16 July 2022; code: CO2161a; US Biomax, Inc., Derwood, MD, USA) to detect the intensity and distribution of EPLIN and HSP60. In brief, IHC assay was carried out as follows. After rehydration and washing, slides were blocked with 10% horse serum for an hour, then were incubated with primary antibodies (EPLIN: monoclonal, mouse, 1:500, sc-136339; HSP60: monoclonal, mouse, 1:500, Santa Cruz Biotechnology, Inc. Dallas, TX, USA) overnight. Secondary antibody was applied after stringent washing followed by incubating with avidin-biotin complex (ABC) reagent (Vector Laboratories, Inc., Newark, CA, USA) and 3′3 diaminobenzidine (DAB) substrate (5 mg/mL) to develop staining. Counterstaining was performed with Gill’s haematoxylin (Vector Laboratories Inc., CA, USA) and rehydrated through a series of graded alcohols and cleared in xylene (Fisher Scientific, Leicestershire, UK). Analysis of IHC staining was performed by scoring intensity and distribution of targeted proteins by two researchers. 

### 4.8. Exploration of Rectum Adenocarcinoma Datasets in Kaplan–Meier Plotter 

An online platform, Kaplan–Meier Plotter was carried out to investigate implication of EPLIN, Her2 and HSP60 on OS and RFS of rectum adenocarcinoma patients. Data and graphs were assessed from https://kmplot.com/analysis/index.php?p = service&cancer = pancancer_rnaseq (accessed on 1 October 2022). 

### 4.9. Generation of EPLINα Overexpression Cell Lines Using Electroporation-Based Transfection

Overexpressed EPLIN models were generated by using a plasmid DNA which contains the open reading frame of a sequence of EPLINα or Stuffer300 control and a puromycin resistant sequence (VectorBuilder Inc., Chicago, IL, USA) via electroporation-based transfection. In brief, 5 μL of plasmid DNA was mixed with 1 mL of cell mixture containing 1 × 10^6^ of cells with antibiotic-free medium in an electroporation cuvette (Geneflow Ltd., Litchfield, UK). After incubating for 5 min, the solution was applied to the BioRad Cell Pulser Xcell electroporation system (BioRad Laboratories, Hertfordshire, UK) and was pulsed at 290 V and 1000 μF. Cells went through selection with culture medium that contains 2 μg/mL puromycin for 72 h and were subsequently kept culturing in medium that contains 0.2 μg/mL puromycin. 

### 4.10. Generation EPLIN Knockdown Cell Lines Using shRNA-Based Transfection

EPLIN shRNA plasmid (sc-60593-SH) was used to knock down expression of EPLIN in cell lines from colorectal cancer with shRNA transfection reagent (Santa Cruz Biotechnology, Inc., Dallas, TX, USA), following instruction from the manufacturer. After selecting with medium that contains 2 μg/mL puromycin, cells were cultured in medium that contains 0.2 μg/mL puromycin to maintain the effect of transfection. 

### 4.11. Generation HSP60 Knockdown Cell Lines Using siRNA-Based Transfection

HSP60 siRNA (sc-29351) was utilised to silence expression of HSP60 in cell lines from colorectal cancer using siRNA transfection reagent (Santa Cruz Biotechnology, Inc., Dallas, TX, USA). The transfection was performed following instruction from the manufacturer. 

### 4.12. Thiazolyl Blue Tetrazolium Bromide (MTT) Based Cellular Growth Assay

MTT based cellular growth assays were performed to investigate EPLIN’s impact on cellular growth. In brief, 3 × 10^3^ cells from each cell models were seeded onto three 96-well plates at triplicate, then were incubated at 37 °C with 5% CO_2_. At Day 1, Day 3 and Day 5, each well of the 96-well plate was supplemented with 22 μL of 5 mg/mL MTT solution (Sigma- Aldrich Co., Poole, Dorset, UK) and was incubated for 4 h at 37 °C with 5% CO_2_. After incubation, medium was aspirated and 100 μL of Dimethyl sulfoxide (DMSO) (Sigma-Aldrich Co., Poole, Dorset, UK) was added into each well. The plate was allowed to be incubated for 10 min at 37 °C with 5% CO_2_. Absorbance was detected in a LT4500 plate reader (Wolf Laboratories, York, UK) at 540 nm. 

### 4.13. Matrigel Adhesion Assay

Matrigel adhesion assay was performed to investigate cellular adhesive function. In brief, Matrigel (BD Biosciences, Oxford, UK) was diluted in serum-free medium (SFM) to the concentration of 0.05 mg/mL. One well of 96-well was coated with 5 μg of Matrigel solution and was allowed to dry in oven at 55 °C before use. After rehydration for 30 min with SFM, each precoated well was seeded by 4 × 10^4^ cells from each cell models at 6 repeats, then was incubated for 40 min at 37 °C with 5% CO_2_. Medium was discarded and each well of the 96-well plate was fixed with 100 μL of 4% formalin solution for 10 min followed by staining with 100 μL of 0.5% crystal violet solution for 10 min. Once the plate was dry. Photos of 4 random areas of each well were taken under a Leica DM IRB microscope (X20) with the Leica LAS EZ system (Leica Microsystems (UK) Ltd., England, UK). Image J (National Institute of Mental Health, Bethesda, Maryland, USA) was utilised to count cells and statistical analysis was performed using GraphPad. 

### 4.14. Matrigel Invasion Assay 

Matrigel invasion assay was carried out to examine cellular invasion. In brief, Matrigel was diluted with SFM to the concentration of 0.5 mg/mL. Then, 50 μg was used to coat a trans-well insert which was placed in a 24-well plate and contains 8.0μm pores. After the inserts were dry and rehydrated with SFM for 30 min. 2.5 × 10^4^ cells from each model were seed into the upper chamber and were incubated for 72 h at 37 °C with 5% CO_2_. Medium was then discarded carefully before invaded cells at the underside of the insert was fixed with 4% formalin solution for 10 min. Cells were then stained with 0.5% crystal violet solution for 10 min and were allowed to dry at room temperature. 4 random fields were chosen under a Leica DM IRB microscope (X20) and photos of such areas were taken by the Leica LAS EZ system. ImageJ was applied to count invaded stained cells and statistical analysis was performed using GraphPad. 

### 4.15. Electric Cell-Substrate Impedance Sensing (ECIS) Based Cell Migration Assay

ECIS assay was used to measure impedance after wounding to investigate cell’s ability to migrate across the wound. 20 × 10^3^ cells from each model were seeded into the 96-well ECIS W961E electrode arrays in 5–6 repeats. The arrays were then placed on the placed on the ECIS Ztheta instrument (Applied Biophysics Ltd., Troy, NJ, USA). Wounding (2000 mA for 20 s) was initiated after incubating for 5 h at 37 °C. Impedance was recorded from 1000 to 64 × 10^3^ Hz by the ECIS system for 10–24 h. Data was analysed using the ECIS software.

### 4.16. Kinexus^TM^ Antibody-Based Protein Microarray 

Kinex^TM^ KAM-880 antibody-based protein microarray (Kinexus Bioinformatics Ltd., Vancouver, BC, Canada) was performed on two pairs of patient’s protein (normal: ID126 & 128; tumour: ID127 & 129) to investigate potential interacting partners. In brief, protein samples were extracted and quantified before they were precipitated with EPLIN antibody (immunoglobulin G (IgG) monoclonal, Sc- 136399, Santa Cruz Biotechnology, Inc. Dallas, TX, USA). Samples were then sent to Kinexus Bioinformatics Ltd., and applied on the arrays to test with 877 antibodies.

### 4.17. Cytotoxicity Assays

Cytotoxicity assays were utilised to investigate implication on drug resistance. In brief, 96-well plates were precoated with 100 μL of serial diluted chemotherapeutic or EGFR/Her2 targeted therapeutic agents. 100 μL cell solution which contained 5 × 10^3^ cells from each cell models were seeded into the 96-well plate in 3 repeats for each concentration. After incubating for 72 h at 37 °C with 5% CO_2_, medium was discarded and the plates were fixed with 100 μL of 4% formalin solution for 10 min. Cells were then stained with 100 μL of 0.5% crystal violet solution, washed and were allowed to dry at room temperature. After the plates were dry, 100 μL of 10% acetic acid was added into each well and incubated for 10 min to extract crystal violet stain. Absorbance was read in a LT4500 plate reader at 595 nm.

### 4.18. Statistical Analysis 

GraphPad (Prism 8) (GraphPad Software, San Diego, CA, USA), Minitab (Minitab Ltd. Coventry, UK) and SPSS version 26 (IBM, Armonk, New York, NY, USA) were used for statistical analysis in this study.

## Figures and Tables

**Table 1:** Transcript expression profile of EPLIN in comparison to clinical pathological information of the colorectal cancer clinical cohort.

Characteristic	Sample Number (n)	Median Transcript Expression	Q1	Q3	*p* Value
Tumour	94	137	3	9745	
Normal	80	6515	140	1,352,500	<0.001 ^a^
Differentiation					0.114 ^b^
High	2	10,848	*	*	
Moderate	54	6428	0	7222	
Low	14	21,964	27	15,319	
TNM stage					0.051 ^b^
TNM1	9	4	0	11,007	
TNM2	30	26	1	8208	
TNM3	26	103	5	1800	
TNM4	6	21,580	1633	37,877	
T stage					0.879 ^b^
T1	2	13,602	*	*	
T2	10	49	0	16,073	
T3	40	73	2	7774	
T4	18	140	13	5039	
Dukes stage					0.382 ^b^
Dukes A	7	95	0	21,600	
Dukes B	33	10	1	7498	
Dukes C	32	146	11	13,004	
Nodal involvement					0.517 ^b^
N0	39	16	1	8050	
N1	16	162	6	13,004	
N2	15	142	21	2130	
Metastasis					0.5910 ^a^
No metastasis	50	35	3	8208	
Distant metastasis	19	95	0	9325	
Incidence					0.5249 ^a^
Disease free	35	29	3	8050	
With incidence	23	95	0	1197	
Recurrence					0.9494 ^a^
No Recurrence	58	29	2	7222	
Local Recurrence	7	139	0	235	
Survival					0.9107 ^a^
Alive	36	32	4	1849	
Died	22	103	1	9374	
Invasiveness					
Non invasive	50	17.2			
Invasive	26	182			0.0323 ^a^

* ^a^ Mann-Whitney; ^b^ Kruskal-Wallis ANOVA on RANKS.

**Table 2:** Analysis of EPLIN staining by immunohistochemistry in the colorectal cancer TMA (CO2161a).

	Total Number	Intensity		Statistical Significance	
		Negative to Weak (0–1)	Moderate to Strong (2–3)	Chi Value	*p*
Pathology					
Normal tissue	8	4	4		
Adenocarcinoma	175	104	71	0.281	0.596 ^a^
Mucinous adenocarcinoma	30	24	6	2.931	0.0869 ^a^
Signet ring cell carcinoma	3	2	1	0.2444	0.621 ^a^
Stage				1.830	0.6085 ^b^
I	18	10	8		
II	115	70	45		
III	70	48	22		
IV	4	3	1		
Differentiation Code				2.227	0.3284 ^c^
Grade1	33	17	15		
Grade2	98	65	33		
Grade3	56	37	18		

^a^ Compared with the normal tissue group; ^b^ Overall chi-square analysis among stage groups; ^c^ Overall chi- square analysis among differentiation groups.

**Table 3:** Quantitative report of proteins that interact with EPLIN (Partly). Globally normalised intensity, intensity of each tested antibody was normalised by all the net signal median values from the samples. %CFC, percentage changes of normalised intensity from normal samples compared with tumour samples. Z-ratio, Z score differences were separated by standard deviation for the comparison. Priority leads were selected as %CFC ≥ 50; SUM of %Error Ranges <0.75 × %CFC value; At least one Globally Normalized intensity value ≥ 1500.

Target Protein Name	Antibody Codes	Globally Normalized Normal	Globally Normalized Tumour	%CFC (Tumour from Normal)	Z-ratio (Tumour, Normal)	Best Leads
ErbB2	NK054-2	1076	3148	192	3.96	Priority
B-Raf	NK156-4	18,354	36,112	97	3.44	Priority
STAT5B	NN106	1523	3849	152	3.43	Priority
Hsp90b	NN165	3604	5987	67	2.73	Priority
MAPK9 (JNK2)	NK088-2	2265	4632	104	2.65	Priority
MAPK7 (ERK5)	NK206-3	3371	5149	53	2.29	Priority
Hsc70	NN054-2	8506	14,370	69	1.97	Priority
PDGFRa	PK758	3078	4976	61	1.79	Priority
Hsp60	NN059-2	15,137	24,097	59	1.76	Priority
Hsp60	NN059-3	1266	1977	56	1.65	Priority
PDGFRb	NK243-3	5131	7989	55	1.66	Priority
PKA Ca/b	PK067	17,981	27,421	52	1.60	Priority
JNK1	NK217	425	220	−48	−2.42	Priority
STAT3	PN082-1	2689	1220	−55	−2.89	Priority
STAT6	NN107	714	1246	74	2.05	Possible
HspBP1	NN063	558	1169	110	4.16	Possible
ERK1	NK055-2	16,878	32,201	91	3.29	Possible

**Table 4:** Transcript expression profile of HSP60 in comparison to clinical pathological information of the clinical cohort.

Characteristic	Sample Number (n)	Median Transcript Expression	Q1	Q3	*p*-Value
Tumour	94	0.45	0	39.5	
Normal	80	0.05	0.01	9.10	0.0097 ^a^
Differentiation					0.562 ^b^
High	2	27	*	*	
Moderate	54	0.1	0	29.8	
Low	14	1.6	0.1	54.3	
TNM stage					0.045 ^b^
TNM1	9	53.9	13.5	255.4	
TNM2	30	0.1	0	5.3	0.0124 ^a^
TNM3	26	0.1	0	4.3	0.0201 ^a^
TNM4	6	32.9	13.3	93.1	0.5169 ^a^
T stage					0.033 ^b^
T1	2	152	*	*	
T2	10	54.3	0.1	275.7	
T3	40	0.09	0.01	6.38	
T4	18	0.4	0	19.6	
Dukes stage					0.235 ^b^
Dukes A	7	36.2	0.1	242.6	
Dukes B	33	0.2	0	16	
Dukes C	32	0.2	0	32.6	
Nodal involvement					
Negative	39	0.5	0	38.8	
N1	16	0.3	0	48.8	0.9114 ^a^
N2	15	0.1	0	11.2	0.6354 ^a^
All node positive	0.2	0.2	0	27.3	0.8369 ^a^
Metastasis					
No metastasis	50	0.2	0	37	
Distant metastasis	19	0.17	0.01	2.13	0.3634 ^a^
Incidence					
Disease free	35	1	0	53.9	
With incidence	23	0.11	0.01	1.16	0.0845 ^a^
Recurrence					
No Recurrence	58	0.2	0	31.6	
Local Recurrence	7	0.459	0.106	1.165	0.8888 ^a^
Survival					
Alive	36	0.2	0	38.1	
Died	22	0.09	0.01	2.73	0.1914 ^a^
					
Non invasive	50	0.2	0	25.1	
Invasive	26	0.2	0	43.4	0.8927 ^a^

* ^a^ Mann-Whitney; ^b^ Kruskal-Wallis ANOVA on RANKS.

**Table 5:** Transcript expression profile of Her2 in comparison to clinical pathological information of the cohort.

Characteristic	Sample Number (n)	Median Transcript Expression	Q1	Q3	*p*-Value
Tumour	94	0.4103	0.066	1.352	
Normal	80	0.0043	0.0002	0.02	<0.001 ^a^
Differentiation					0.379 ^b^
High	2	0.553	*	*	
Moderate	54	0.810	0.186	1.591	
Low	14	0.302	0.055	1.284	
Moderate and low	67	0.553	0.149	1.560	
TNM stage					0.492 ^b^
TNM1	9	0.29	0.05	1.52	
TNM2	30	0.874	0.224	1.561	
TNM3	26	0.393	0.114	1.616	
TNM4	6	0.118	0.003	1.211	
T stage					0.571 ^b^
T1	2	0.1217	*	*	
T2	10	0.35	0.19	1.31	
T3	40	0.72	0.139	1.42	
T4	18	1.046	0.122	1.887	
Dukes stage					0.548 ^b^
Dukes A	7	0.77	0	1.94	
Dukes B	33	0.846	0.224	1.309	
Dukes C	32	0.363	0.048	1.503	
Nodal involvement					
N0	39	0.784	0.178	1.477	
N1	16	0.298	0.144	1.591	0.5942 ^a^
N2	15	0.413	0.009	1.355	0.6065 ^a^
N1&2	0.2	0.373	0.073	1.544	0.5100 ^a^
Metastasis					
No metastasis	50	0.413	0.0688	1.313	
Distant metastasis	19	1.123	0.176	2.088	0.1767 ^a^
Incidence					
Disease free	35	0.843	0.243	1.616	
With incidence	23	1.153	0.17	2.088	0.6385 ^a^
Recurrence					
No Recurrence	58	0.431	0.142	1.37	
Local Recurrence	7	1.248	0.95	2.643	0.1649 ^a^
Survival					
Alive	36	0.902	0.226	1.645	
Died	22	1.141	0.208	1.898	0.666 ^a^
					
Non invasive	50	0.879	0.16	1.55	
Invasive	26	0.376	0.032	1.402	0.8927 ^a^

* ^a^ Mann-Whitney; ^b^ Kruskal-Wallis ANOVA on RANKS.

**Table 6:** Analysis of HSP60 staining in the colorectal cancer TMA (CO2161a).

	Total Number	Intensity		Statistical Significance	
		Negative to Weak (0–1)	Moderate to Strong (2–3)	Chi Value	*p*
Pathology					
Normal tissue	8	8	0		
Adenocarcinoma	175	92	83		
Mucinous adenocarcinoma	30	26	4	12.19	0.0005 ^a^
Signet ring cell carcinoma	3	3	0		
Stage					
I	18	11	7		
II	115	68	47	0.1591	0.8736 ^b^
III	70	38	32	0.2703	0.6031 ^b^
IV	4	3	1	0.2728	0.6014 ^b^
Differentiation Code				7.074	0.0291 ^c^
Grade1	33	19	14		
Grade2	98	46	52		
Grade3	55	38	17		

^a^ Compared with adenocarcinoma group; ^b^ Compared with Stage I group; ^c^ Overall chi-square analysis among differentiation groups.

**Table 7:** Cox regression multivariate analysis on Cardiff clinical cohort. Combination of EPLIN, HSP60 and Her2 were identified to be a significant predictor affecting OS on CRC patients (*p* = 0.024).

	Significance	Hazard Ratio
Invasion	0.215	2.685
Treatment	0.581	1.267
Location	0.098	0.540
Dukes	0.063	0.096
Stage	0.231	2.249
TNM	0.432	2.155
Node	0.754	0.634
Differentiation	0.381	0.488
EPLIN, HSP60 & Her2	0.024	5.461

**Table 8:** Cox regression multivariate analysis of RFS of CRC patients in the clinical cohort. Dukes stage was found to be a significant predictor (*p* = 0.021) as well as TNM stage (*p* = 0.04). Additionally, the combination of EPLIN, HSP60 and Her2 expression was also found to be a significant predictor to affect CRC patients’ RFS (*p* = 0.049).

	Significance	Hazard Ratio
Invasion	0.141	2.933
Treatment	0.585	1.241
Location	0.249	0.676
Dukes	0.021	0.065
Stage	0.698	1.267
TNM	0.040	5.980
Node	0.210	0.205
Differentiation	0.094	0.277
EPLIN, HSP60 & Her2	0.049	2.929

**Table 9:** Spearman’s correlation between EPLIN, Hers and HSP60 in tumour samples from the clinical cohort. * represents *p* value (two-tailed) < 0.05, ** represents *p* value (two-tailed) < 0.01.

Name		Her1 (Tumour)	Her2 (Tumour)	Her3 (Tumour)	Her4 (Tumour)	HSP60 (Tumour)
EPLIN (Tumour)	Correlation	0.09	−0.152	−0.272 *	0.191	0.488 **
	Number	91	92	75	94	93
	*p* value	0.394	0.148	0.018	0.065	<0.01
Her1 (Tumour)	Correlation	1	0.179	−0.078	0.220 *	0.406 **
	Number	91	89	75	91	90
	*p* value	*	0.093	0.507	0.036	<0.01
Her2 (Tumour)	Correlation	0.179	1	0.248 *	−0.029	0.012
	Number	89	92	73	92	91
	*p* value	0.093	*	0.034	0.784	0.909
Her3 (Tumour)	Correlation	−0.078	0.248 *	1	−0.230 *	−0.179
	Number	75	73	75	75	74
	*p* value	0.507	0.034	*	0.047	0.127
Her4 (Tumour)	Correlation	0.220 *	−0.029	−0.230 *	1	0.248 *
	Number	91	92	75	94	93
	*p* value	0.036	0.784	0.047	*	0.017
HSP60 (Tumour)	Correlation	0.406 **	0.012	−0.179	0.248 *	1
	Number	90	91	74	93	93
	*p* value	<0.01	0.909	0.127	0.017	*

**Table 10:** IC50 values of the cytotoxicity assays on RKO&HRT18 cell models.

	5-Fluorouracil	Docetaxel	Oxaliplatin	AG825	Neratinib
**RKO cell models**					
RKO-WT	10.66 μM	0.27 nM	4.01 μM	34.90 μM	0.55 μM
RKO-KD-EPLIN	23.44 μM	0.24 nM	3.28 μM	12.87 μM	0.25 μM
RKO-KD-HSP60	15.39 μM	0.24 nM	3.68 μM	9.87 μM	0.66 μM
RKO-KD-EPLIN/HSP60	13.01 μM	0.22 nM	7.89 μM	23.09 μM	0.54 μM
**HRT18 cell models**					
HRT18-WT	8.97 μM	0.26 nM	8.97 μM	108.10 μM	0.34 μM
HRT18-KD-EPLIN	11.47 μM	0.15 nM	0.31 μM	51.21 μM	0.05 μM
HRT18-KD-HSP60	13.73 μM	0.047 nM	0.54 μM	54.05 μM	0.03 μM
HRT18-KD-EPLIN/HSP60	13.22 μM	0.098 nM	64.04 μM	46.58 μM	0.096 μM

## Data Availability

Data presented in this publication can be obtained from the lead author upon reasonable request.
